# Professional Social Media Usage and Work Engagement Among Professionals in Finland Before and During the COVID-19 Pandemic: Four-Wave Follow-Up Study

**DOI:** 10.2196/29036

**Published:** 2021-06-15

**Authors:** Reetta Oksa, Markus Kaakinen, Nina Savela, Jari J Hakanen, Atte Oksanen

**Affiliations:** 1 Faculty of Social Sciences Tampere University Tampere Finland; 2 Institute of Criminology and Legal Policy, Faculty of Social Sciences University of Helsinki Helsinki Finland; 3 Work Ability and Working Careers Finnish Institute of Occupational Health Helsinki Finland

**Keywords:** COVID-19, engagement, mental health, moderator, predictor, psychological distress, social media, social support, support, task resources, usage, work engagement

## Abstract

**Background:**

The COVID-19 pandemic has changed work life profoundly and concerns regarding the mental well-being of employees’ have arisen. Organizations have made rapid digital advancements and have started to use new collaborative tools such as social media platforms overnight.

**Objective:**

Our study aimed to investigate how professional social media communication has affected work engagement before and during the COVID-19 pandemic and the role of perceived social support, task resources, and psychological distress as predictors and moderators of work engagement.

**Methods:**

Nationally representative longitudinal survey data were collected in 2019-2020, and 965 respondents participated in all 4 surveys. Measures included work engagement, perceived social support and task resources, and psychological distress. The data were analyzed using a hybrid linear regression model.

**Results:**

Work engagement remained stable and only decreased in autumn 2020. Within-person changes in social media communication at work, social support, task resources, and psychological distress were all associated with work engagement. The negative association between psychological distress and work engagement was stronger in autumn 2020 than before the COVID-19 outbreak.

**Conclusions:**

The COVID-19 pandemic has exerted pressure on mental health at work. Fostering social support and task resources at work is important in maintaining work engagement. Social media communication could help maintain a supportive work environment.

## Introduction

The rapid spread of the COVID-19 pandemic has affected our lives and work profoundly [1,2]. The COVID-19 pandemic has pressurized organizations to make a rapid digital leap to remote work and thus challenged and cultivated employees’ well-being [3,4]. In Europe, 37% of the employees began working remotely in March and April 2020, with Finland having the largest proportion of remote workers (59%) [3]. In 2019, prior to the COVID-19 pandemic, only 23% of people in Finland worked remotely from home or other locations regularly, and 14% did so occasionally; therefore, the leap has been enormous [5].

In remote work conditions during the COVID-19 pandemic, the use of digital tools and social media platforms has increased at work for information and document creation, sharing, and exchange and for video meetings and discussions [6]. These tools are often used for both work and nonwork purposes among colleagues and have been found to enhance ways of working, foster innovation, allow for learning new skills, enhance performance, foster social relationships and social support, organizational identification, enable job satisfaction, and work engagement [6-11]. However, there is currently a lack of research on their role during the pandemic.

Work engagement, a key positive motivational state of well-being at work, is a comprehensive and enduring positive mental state that employees experience at their workplace and consists of three dimensions: vigor (ie, high energy levels, mental resilience, and persistence), dedication (ie, a sense of significance and pride), and absorption (ie, deep concentration on work and challenges detaching from work) [12,13]. Work engagement among employees in Finland was favorable before the COVID-19 crisis: 63% experienced vigor, 64% experienced dedication, and 56% experienced absorption in their work often or always [5].

According to job demands-resources model, work engagement is particularly driven by job resources, which are positive psychological, physical, social, and organizational characteristics of work, such as a good organizational climate and social support from colleagues and supervisors, which help employees accomplish work goals and foster learning and personal growth [13,14]. Social support defined as emotional, informational, and instrumental support, which describes not only the functional importance of relationships, but also the quality of those relationships and social belonging, can be a great reciprocal resource, for example, in coping stress and enhancing self-efficacy [15-18]. Engaged employees are more likely to be proactive and productive in their work [19]. Furthermore, autonomy, possibility to engage in meaningful work, and opportunities to leverage their strengths and experience at work are important factors influencing employee engagement [20-21].

Based on the Conservation of Resources (COR) theory, people tend to obtain and protect valuable resources, and loss of resources plays a significant role in the development of psychological stress [22]. Work engagement, as an energetic resource that employees may possess, should be a key priority in organizations, as it can increase life satisfaction and can prevent employees from psychological distress, depression, anxiety, sickness absenteeism, and burnout [23-26]. Furthermore, work engagement has been associated with healthy cardiac autonomic activity and a low likelihood of disability pensions [27,28]. Notably, high levels of work engagement have also been associated with increased short-term psychological distress and with decreased psychological stress over time [29]. At the societal level, work engagement predicts less unemployment in the general population [27].

The COVID-19 pandemic, along with its associated increase in digital and remote work, has potentially transformed ways of working for good [30]. Prior literature indicates that in the digital work environment, employees appreciate the opportunity to influence their work and enjoy the freedom and flexibility to complete their tasks; thus, they experience agency and higher self-esteem [31]. Resources such as support from managers received on social media can prevent work-related psychological distress [32,33]. Recent studies on the COVID-19 pandemic have reported that personal resilience and organizational and social support can sustain employee well-being and prevent anxiety [34]. Low supervisor support can, in turn, predict lower well-being, including stress, exhaustion, and burnout [35]. Furthermore, a study on adults in the United States reported that psychological distress increased from 3.9% in 2018 to 13.6% in 2020 during the COVID-19 pandemic [36]. Indeed, employees in the medical field have reported increased psychological distress and decreased well-being owing to heightened demands and workloads [34,37].

According to the COR theory [22] resource gains (such as supervisor support) in themselves have only a modest effect on well-being, but instead acquire saliency in the context of resource loss. Thus, prolongation of the COVID-19 pandemic can be considered a resource threat for employees. It can be argued that perceived social support and task resources have been particularly important in autumn 2020 as social distancing policies had been implemented since spring 2020 [1,2], and normal social interactions and working practices have been highly limited for a prolonged time. Among the basic psychological needs, particularly relatedness (lack of social contacts) and competence (eg, reduced possibilities to effectively bring about desired effects and outcomes) have been affected [38].

Social media communication at work has increased during the COVID-19 pandemic [6], and prior evidence has shown that work-related social media communication can enhance occupational resources such as social support and organizational identification and moreover work engagement [8]. However, previous studies have also indicated that psychological distress is associated with decreased work engagement before [39] and during the COVID-19 pandemic in spring 2020 [40]. Thus far, little is known about the longitudinal associations between professional social media communication and work engagement or how professional social media communication has affected work engagement and employees’ mental well-being during the COVID-19 pandemic.

This longitudinal study analyzed changes in work engagement among employees in Finland before and during the COVID-19 pandemic. Our study investigated whether changes in social media communication at work, perceived social support, task resources, and psychological distress are related to changes in employees’ work engagement, especially at a time of a prolonged pandemic. We proposed the following hypotheses: (1) increased social media communication predicts an increase in work engagement; (2) increased perceived social support and task resources at work predict an increase in work engagement; (3) increased psychological distress predicts decreased work engagement; and (4) the association between work engagement and (i) social media communication, (ii) social support, and (iii) psychological distress have been stronger during rather than before the COVID-19 pandemic.

## Methods

### Participants and Procedure

Data from a 4-timepoint longitudinal survey on social media usage at work in Finland from 2019 to 2020 were acquired to represent the working population in Finland. The first survey was conducted in March to April 2019 (timepoint 1 [T1]; n=1817). The participants were recontacted in September to October 2019 (timepoint 2 [T2]; n=1318), March to April 2020 (timepoint 3 [T3]; n*=*1081), and September to October 2020 (timepoint 4 [T4]; n*=*1152). The fourth survey was sent to all original respondents, whereas the third survey was sent only to those who had responded to the second survey.

The final sample in this study (n=965; 45.08% female; mean age 44.97 years, SD 11.36 years) included respondents who answered all 4 surveys, and the response rate was 53.11%. We found no major bias when conducting nonresponse analyses and when comparing the sample with official census figures of the working population in Finland [8]. The sample encompassed all major occupational fields and covered all prominent areas of Finland [6]. Analyses focused on employees of working age (18-66 years) and those respondents who remained employed (n=868). Only those respondents who finished the whole survey were included in the final data set. The survey study involved no ethical issues according to the assessment of the Academic Ethics Committee of Tampere region in Finland. The survey was conducted in Finnish, and participation was voluntary. The research group designed the survey and collected data in collaboration with Norstat, whose web-based research panel was used to recruit participants.

### Measures

#### Work-Related and Nonwork-Related Social Media Communication

We measured the frequency of social media usage for work-related communication by asking the question, “How often do you use social media to keep in touch with your colleagues or work community regarding work-related matters (eg, sharing information or agreeing on timetables)?” We measured the frequency of social media usage for nonwork-related communication by asking the question, “How often do you use social media to keep in touch with your colleagues or work community regarding nonwork-related matters?” Possible answers were 0=“I don’t use it,” 1=“less than weekly,” 2=“weekly,” 3=“daily,” and 4=“many times a day.” Both social media communications were measured at every time point; that is, every 6 months.

#### Work Engagement

Work engagement is most often measured using the Utrecht Work Engagement Scale (UWES) [41]. The 9-item version of this scale, UWES-9, is used most often owing to its construct validity [42]. Example questionnaire items include the following: “At my work, I feel that I am bursting with energy” and “I feel happy when I am working intensely.” Responses are scored on a scale ranging from 0=“never” to 6=“always/every day.” All 3 dimensions of the UWES were summed up to create a composite variable with a range of 0-54 and the Cronbach α coefficient was measured for all timepoints, ranging from .95 to .96. Work engagement was measured at every timepoint; that is, every 6 months.

#### Perceived Social Support

Perceived social support at work was measured using 4 questions on social support received from colleagues, supervisors, and the work community in general. These questions originate from the second version of the Copenhagen Psychosocial Questionnaire (Multimedia Appendix 1) [43], and they have been previously validated as a measure for social support at work [8]. Scores associated with these 4 items were summed to obtain a composite variable with a range of 4-20. Higher figures indicate higher perceived social support. The scale showed high reliability (Cronbach α=.74-.79). Perceived social support was measured at every timepoint; that is, every 6 months.

#### Task Resources

Task resources were measured using 4 questions from the work organization and job content dimension of the second version of the Copenhagen Psychosocial Questionnaire (Multimedia Appendix 2) [43]. Scores associated with the 4 questions were summed to obtain a composite variable with a range of 4-20. The scale showed adequately high internal consistency (Cronbach α=.67-.69). Task resources were measured at every time point; that is, every 6 months.

#### Psychological Distress

We measured psychological distress using the 12-item General Health Questionnaire [44]. Example questions included the following: “Have you recently felt constantly under strain?” and “Have you recently felt capable of making decisions about things?” Scores associated with all items were summed to obtain a composite variable with a range of 0-36. Higher scores indicate higher psychological distress. The scale showed high reliability (Cronbach α=.89-.92) between measurement points. Psychological distress was measured at every timepoint; that is, every 6 months.

#### Background Variables

Sociodemographic variables considered herein included age, gender, and education. All background variables were assessed at every timepoint; that is, every 6 months.

### Statistical Analyses

As descriptive statistics, we expressed data as mean (SD) values for continuous study variables and frequencies and proportions for categorical variables (Tables 1 and 2). In addition, SD values between measurements were calculated for the within-person–level variables. We also assessed correlations among our study variables measured at different timepoints (Multimedia Appendix 3).

For all our hypotheses, we analyzed whether the within-person variation in social media communication, perceived social support, task resources, and psychological distress predicted changes in work engagement. We tested our hypotheses using a hybrid (or within-between) linear regression model [45]. This method decomposes the association between the dependent variables and time-variant independent variables into within-person and between-person effects. This is carried out by adding the individual means of dependent variables (between-person effects) and individual deviations from the person means (within-person effects) into the model simultaneously. Between-person effects are then estimated as associations between the individual means of the dependent and independent variables. Within-person effects are estimated as associations between the dependent variable and the observed deviation from the individual means. Thus, the between-person effects describe static differences between individuals, whereas within-person effects describe a dynamic relationship between the timely fluctuations in both the dependent and independent variables.

**Table 1:** Descriptive statistics of the study variables: continuous variables.

Variables		Time					Within-person differences, SD
	Range	T1, mean (SD)	T2, mean (SD)	T3, mean (SD)	T4, mean (SD)		
Work engagement	0-54	38.78 (12.13)	39.08 (12.15)	39.29 (11.64)	38.42 (12.04)	5.35	
Work-related social media communication	0-4	1.27 (1.21)	1.31 (1.19)	1.52 (1.21)	1.51 (1.25)	0.69	
Nonwork-related social media communication	0-4	1.16 (1.06)	1.10 (0.99)	1.24 (1.06)	1.18 (1.01)	0.59	
Social support	4-20	14.65 (2.86)	14.56 (2.87)	14.68 (2.91)	14.65 (3.01)	1.49	
Task resources	4-20	13.89 (2.76)	13.98 (2.74)	14.03 (2.63)	13.90 (2.70)	1.31	
Psychological distress	12-48	24.89 (6.21)	24.14 (5.60)	24.26 (5.29)	24.19 (5.53)	3.32	
Age in T1 (years)	18-64	43.52 (10.86)	N/A^a^	N/A	N/A	N/A	

^a^N/A: not applicable.

**Table 2:** Descriptive statistics of the study variables: categorical variables.

Variables	Values	
	Coding	Number of participants, n (%)
Females^a^	0/1	379 (43.7)
Basic education	0/1	26 (3.0)
Secondary degree	0/1	429 (49.4)

^a^Number of participants at each time point (T1-T4)=868; total number of observations (T1+T2+T3+T4)=3472.

Our analysis proceeded in 2 steps. Model 1 included all our within-person and between-person main effects and a random intercept. For work-related and nonwork-related social media communication, perceived social support, task resources, and psychological distress, the effects were estimated as within- and between-person effects. For time, we estimated only within-person effects. Time was included as binary variables (T2-T4) with T1 as a reference category. Gender, age, and education at T1 were added to the model as between-person variables, as they varied only between persons.

To test our hypothesized moderation effects, within-person interaction terms including work-related and nonwork-related social media communication, perceived social support, task resources, and psychological distress at T4 were added to the model; Schunck [46] has described the estimation of within-person interaction terms. The significant interaction terms (95% CI) are reported in Model 2 in Tables 3 and 4. We report unstandardized regression coefficients (Β), their estimated SE values, significance (*P* value), the variance of random intercept, and a log pseudolikelihood estimate in Tables 3 and 4. For effect size estimates, we reported Cohen *f*^2^ coefficients for all the significant predictors. These coefficients were calculated using the approach described by Selya et al [47] and they can be interpreted as the proportion of explained variance associated with certain independent variables [48].

**Table 3:** Within-between models predicting changes in work engagement with time: fixed effects.

Variables			Model 1					Model 2		
			B (SE)		*P* value		B (SE)			*P* value
Constant			4.84 (3.82)		.21		3.99 (3.89)			.31
**Within-person variables**										
	T2 (reference: T1)	0.08 (0.26)		.75		0.11 (0.26)			.68	
	T3 (reference: T1)	0.08 (0.28)		.77		0.10 (0.28)			.71	
	T4 (reference: T1)	–*0.66*^a^ *(0.29)*		*.02*		2.69 (1.31)			*.04*	
	Work-related social media communication	*0.38 (0.15)*		*.009*		*0.38 (0.15)*			*.01*	
	Nonwork-related social media communication	0.11 (0.17)		.50		0.12 (0.17)			.48	
	Social support	*0.82 (0.09)*		*<.001*		*0.81 (0.09)*			*<.001*	
	Task resources	*0.91 (0.10)*		*<.001*		*0.92 (0.10)*			*<.001*	
	Psychological distress	–*0.28 (0.04)*		*<.001*		–*0.25 (0.04)*			*<.001*	
**Between-person variables**										
	Females	*4.02 (0.54)*		*<.001*		*4.02 (0.54)*			*<.001*	
	Basic education	–1.97 (1.87)		.29		–1.97 (1.87)			.29	
	Secondary degree	–0.11 (0.54)		.84		–0.11 (0.54)			.84	
	Age at T1 (years)	*0.08 (0.02)*		*.003*		*0.08 (0.02)*			*.003*	
	Work-related social media communication	0.44 (0.39)		.26		0.44 (0.39)			.26	
	Nonwork-related social media communication	*1.35 (0.45)*		*.003*		*1.35 (0.45)*			*.003*	
	Social support	*0.72 (0.14)*		*<.001*		*0.72 (0.14)*			*<.001*	
	Task resources	*1.89 (0.15)*		*<.001*		*1.89 (0.15)*			*<.001*	
	Psychological distress	–*0.57 (0.08)*		*<.001*		–*0.57 (0.08)*			*<.001*	
**Within-level interactions**										
	Psychological distress at T4	N/A^b^		N/A		–*0.14 (0.05)*			*.012*	

^a^Values in italics are significant.

^b^N/A: not applicable.

**Table 4:** Within-between models predicting changes in work engagement with time: random effects.

Variables	Model 1	Model 2
Intercept, variance (95% CI)	52.49 (45.37-60.73)	52.52 (45.40-60.76)
Log pseudolikelihood	–11753.96	–11748.44

## Results

The results of descriptive statistical analysis are shown in Tables 1 and 2. There were no significant changes in work engagement in T1-T3; however, in T4, work engagement decreased (Β=–0.66; *P*=.02) (Table 3). The effect size of this change was low (Cohen *f*^2^<.01). Among the other within-person variables, an increase in work-related social media communication (Β=0.38; *P*=.009), social support (Β=0.82; *P*<.001), and task resources (Β=0.91; *P*<.001) were associated with increased work engagement. Increased psychological distress, in turn, was associated with reduced work engagement (Β=–0.28; *P*<.001). The variance in work engagement was mainly explained by social support (Cohen *f*^2^=.06), task resources (Cohen *f*^2^=.05), and psychological distress (Cohen’s *f*^2^=.04), and the effect size for work-related social media communication was low (Cohen *f*^2^<.01).

Between-person differences in nonwork-related social media communication (Β=1.35; Cohen *f*^2^<.01; *P*=.003), social support (Β=0.72; Cohen *f*^2^<.01; *P*<.001), and task resources (Β=1.89; Cohen *f*^2^=.01; *P*<.001) were positively associated with average work engagement, yet they only explained a marginal share of the variance in work engagement. Between-person differences in psychological distress, in turn, were negatively associated with work engagement (Β=–0.57; *P*<.001). The effect size for this association was low (Cohen *f*^2^<.01). In addition, female gender (Β=4.02; *P*<.001) and age (Β=0.08; *P*=.003) were associated with between-person differences in work engagement. This implies that females reported higher work engagement on average than males, and older respondents also had higher work engagement on average. However, the effect size was low both for gender (Cohen *f*^2^<.01) and age (Cohen *f*^2^<.01).

Among our moderations (model 2), only the interaction effect between T4 and psychological distress was significantly related to work engagement (Β=–0.14; *P*=.012). As expected, the negative association between within-person differences in work engagement and psychological distress was stronger in autumn 2020 (Β=–0.39) than at T1 (Β=–0.25; *P*<.001). However, the overall proportion of the variance in work engagement explained by this interaction was low (Cohen *f*^2^<.01).

## Discussion

### Principal Findings

This study longitudinally investigated how social media communication at work predicts work engagement. Our theoretical and empirical model was based on the job demands-resources model and COR theory and considered the role of social support and task resources at work, along with psychological distress. Our results show that work engagement remained stable and only decreased in autumn 2020. Within-person changes in social media communication at work, social support, task resources, and psychological distress were associated with work engagement. Moreover, work engagement decreased during autumn 2020 when psychological distress had a stronger negative association with work engagement compared to that before the COVID-19 outbreak.

Our findings partly support hypothesis 1 and fully support hypothesis 2, thus demonstrating that more intensive work-related social media communication and higher perceived social support and task resources are associated with higher within-person work engagement. Nonwork-related communication with colleagues, perceived social support, and task resources were associated with between-person work engagement. However, within-person changes in nonwork-related social media communication did not predict changes in work engagement. Women and older people experienced higher work engagement, as reported previously for individuals in Finland and Europe [49,50].

Increased psychological distress was associated with reduced within-person work engagement, thus supporting hypothesis 3. Our results do not support hypotheses 4-i and 4-ii as the associations between work engagement and social media communication, perceived social support, and task resources did not change during the COVID-19 pandemic. The results partly support hypothesis 4-iii because the within-person association between psychological distress and work engagement was stronger during the COVID-19 pandemic (ie, autumn 2020).

### Comparison With Prior Work

Our study is timely and the first one to offer longitudinal evidence regarding internal and external social media communication, both work-related and nonwork-related, in organizations and the related well-being implications, before and during the COVID-19 pandemic. Our findings revealed that work engagement remained considerably state at the onset of the COVID-19 pandemic during spring 2020. Hence, our results provide interesting insights and are in contrast with those of prior studies reporting that major disasters usually provoke stress and reduce resources [22,51]. However, prolonged uncertain situations have detrimental effects on well-being [52], which our results also confirm.

Increased psychological distress was associated with reduced work engagement in the within-person model, which is in line with prior reports on stress and social media use [53,54]. Individuals experienced higher psychological distress and lower work engagement during the autumn 2020, when COVID-19 was already well-known, and the crisis was underway. Therefore, our results contribute to the current literature on crises and the use of information and communication technologies [55,56], which indicate that a continued crisis has a negative influence on employee well-being and provides further knowledge, especially on professional social media use during the COVID-19 pandemic.

The significant role of various job resources in work engagement construction has been established in prior studies and in the context of social media [8,13,14]. Our findings strengthen the role of job resources in boosting work engagement during the pandemic by demonstrating that an increase in perceived social support and task resources fosters within-person and between-person work engagement.

Our findings have practical implications for organizations by demonstrating that work engagement decreased during autumn 2020, while psychological distress was stronger at that timepoint. Employees continued to work under uncertain conditions in autumn 2020 with no certain signs of future relief. Thus, providing mental health support for employees in such situations is crucial. The importance of supervisor support in alleviating employees’ emotional exhaustion and feelings of uncertainty regarding COVID-19 has been previously reported [57], which our findings also emphasize. Furthermore, our results indicate that work-related social media communication is associated with enhanced work engagement, thus explaining within-person variation. Hence, communication with colleagues via social media can also serve as an important job resource that supports employees’ resources and vigor, as well as their dedication to and absorption in their work.

Increased nonwork-related social media communication did not explain within-person variation in work engagement. We observed only between-person differences because those with high nonwork-related social media communication also had a higher level of work engagement on average. Employees who use social media actively for informal communication are also the ones who engage more in their work. This is because when engaged, employees invest energy into their work-related roles and therefore behave more proactively [58] and have higher contextual performance; that is, an individual’s propensity to behave in ways that facilitate the social and psychological context of an organization [59]. Furthermore, the association between informal social media communication and work engagement might be more complex. For example, prior literature has reported that the association between informal social media communication and work engagement is mediated through other factors such as social support and organizational identification [8].

Moreover, increased social support and task resources were related to enhanced within-person and between-person work engagement. The results emphasize the importance of supporting employees in using their expertise, maintaining a sense of meaningfulness, providing possibilities to influence their work content and load, and offering and receiving social support.

### Strengths and Limitations

We used a longitudinal, nationally representative sample, which enabled the analysis of timepoints before and during the COVID-19 crisis and the related effects on well-being, which can regard as one of the strengths of this study. The response rate was high, and our survey included a very limited number of missing observations. The study design with work-related and nonwork-related social media communication was novel, and a similar longitudinal study has not been performed before. The study was conducted with employed people in Finland and did not examine the COVID-19 crisis cross-nationally. Because this was an observational study, the associations reported herein should not be directly interpreted as causal relationships. Some effect sizes were low, but effect sizes for the main results remained significant even though our model was adjusted for a number of factors. This study was also limited to self-reported information.

### Conclusions

Work engagement decreased during autumn 2020 at a time when psychological distress had a stronger negative association with work engagement. Social media communication at work, perceived social support, and task resources were also associated with higher work engagement. Overall, work engagement remained relatively stable during the COVID-19 crisis. However, providing mental health support during a prolonged crisis is crucial for organizations. Moreover, supporting employees’ resources at work is important in maintaining employee work engagement, in which social media communication can be of help.

## Appendix

Multimedia Appendix 1 Copenhagen Psychosocial Questionnaire II Interpersonal relations and leadership dimension.

Multimedia Appendix 2 Copenhagen Psychosocial Questionnaire II Work organization and job contents dimension.

Multimedia Appendix 3 Correlation matrix.

## Abbreviations

COR: Conservation of Resources
UWES: Utrecht Work Engagement Scale

## References

[1] Oksanen A, Kaakinen M, Latikka R, Savolainen I, Savela N, Koivula A (2020). Regulation and Trust: 3-Month Follow-up Study on COVID-19 Mortality in 25 European Countries. JMIR Public Health Surveill.

[2] Kniffin KM, Narayanan J, Anseel F, Antonakis J, Ashford SP, Bakker AB, Bamberger P, Bapuji H, Bhave DP, Choi VK, Creary SJ, Demerouti E, Flynn FJ, Gelfand MJ, Greer LL, Johns G, Kesebir S, Klein PG, Lee SY, Ozcelik H, Petriglieri JL, Rothbard NP, Rudolph CW, Shaw JD, Sirola N, Wanberg CR, Whillans A, Wilmot MP, Vugt MV (2021). COVID-19 and the workplace: Implications, issues, and insights for future research and action. Am Psychol.

[3] (2020). Living, working and COVID-19: First findings – April 2020. Eurofound.

[4] Kestilä L, Härmä V, Rissanen P (2020). Covid19-epidemian vaikutukset hyvinvointiin, palvelujärjestelmään ja kansantalouteen : Asiantuntija-arvio, syksy 2020. Julkari.

[5] Keyriläinen M (2020). Working conditions barometer 2019. Ministry of Economic Affairs and Employment.

[6] Oksanen A, Oksa R, Savela N, Mantere E, Savolainen I, Kaakinen M (2021). COVID-19 crisis and digital stressors at work: A longitudinal study on the Finnish working population. Computers in Human Behavior.

[7] Nisar T, Prabhakar G, Strakova L (2019). Social media information benefits, knowledge management and smart organizations. J Bus Res.

[8] Oksa R, Kaakinen M, Savela N, Ellonen N, Oksanen A (2020). Professional social media usage: Work engagement perspective. New Media & Society.

[9] Waizenegger L, McKenna B, Cai W, Bendz T (2020). An affordance perspective of team collaboration and enforced working from home during COVID-19. Eur J Inf Syst.

[10] Mäntymäki M, Riemer K (2016). Enterprise social networking: A knowledge management perspective. Int J Inf Manage.

[11] Robertson BW, Kee KF (2017). Social media at work: The roles of job satisfaction, employment status, and Facebook use with co-workers. Comput Hum Behav.

[12] Schaufeli W, Salanova M, Gonzales-Roma V, Bakker A (2002). The measurement of engagement and burnout: A two sample confirmative factor analytic approach. J Happiness Stud.

[13] Schaufeli W, Bakker A (2004). Job demands, job resources, and their relationship with burnout and engagement: a multi-sample study. J Organiz Behav.

[14] Hakanen J, Schaufeli W, Ahola K (2008). The Job Demands-Resources model: A three-year cross-lagged study of burnout, depression, commitment, and work engagement. Work & Stress.

[15] Bakker A (2011). An Evidence-Based Model of Work Engagement. Curr Dir Psychol Sci.

[16] Cohen S, Wills T (1985). Stress, social support, and the buffering hypothesis. Psychol Bull.

[17] Ross CE, Cohen S, Syme SL (1986). Social Support and Health. Contemporary Sociology.

[18] Schwarzer R, Knoll N (2007). Functional roles of social support within the stress and coping process: A theoretical and empirical overview. Int J Psychol.

[19] Xanthopoulou D, Bakker A, Demerouti E, Schaufeli W (2011). Work engagement and financial returns: A diary study on the role of job and personal resources. J Occup Organ Psychol.

[20] Kahn W, Fellows S, Dik BJ, Byrne ZS, Steger MF (2013). Employee engagement and meaningful work. Purpose and meaning in the workplace.

[21] Van Wingerden J, Van der Stoep J (2018). The motivational potential of meaningful work: Relationships with strengths use, work engagement, and performance. PLoS One.

[22] Hobfoll S (2001). The Influence of Culture, Community, and the Nested‐Self in the Stress Process: Advancing Conservation of Resources Theory. Appl Psychol.

[23] Innstrand S, Langballe E, Falkum E (2012). A longitudinal study of the relationship between work engagement and symptoms of anxiety and depression. Stress Health.

[24] Schaufeli W, Taris T, van Rhenen W (2008). Workaholism, Burnout, and Work Engagement: Three of a Kind or Three Different Kinds of Employee Well-being?. Appl Psychol.

[25] Schaufeli W, Bakker A, Van Rhenen W (2009). How changes in job demands and resources predict burnout, work engagement, and sickness absenteeism. J Organiz Behav.

[26] Hakanen J, Schaufeli W (2012). Do burnout and work engagement predict depressive symptoms and life satisfaction? A three-wave seven-year prospective study. J Affect Disord.

[27] Hakanen J, Rouvinen P, Ylhäinen I (2021). The Impact of Work Engagement on Future Occupational Rankings, Wages, Unemployment, and Disability Pensions—A Register-Based Study of a Representative Sample of Finnish Employees. Sustainability.

[28] Seppälä P, Mauno S, Kinnunen M, Feldt T, Juuti T, Tolvanen A, Rusko H (2012). Is work engagement related to healthy cardiac autonomic activity? Evidence from a field study among Finnish women workers. J Posit Psychol.

[29] Shimazu A, Schaufeli W, Kubota K, Watanabe K, Kawakami N (2018). Is too much work engagement detrimental?. PLoS One.

[30] Davison RM (2020). The Transformative Potential of Disruptions: A Viewpoint. Int J Inf Manage.

[31] Dittes S, Richter S, Richter A, Smolnik S (2019). Toward the workplace of the future: How organizations can facilitate digital work. Business Horizons.

[32] Charoensukmongkol P (2014). Effects of support and job demands on social media use and work outcomes. Comput Hum Behav.

[33] Oshio T, Inoue A, Tsutsumi A (2018). Associations among job demands and resources, work engagement, and psychological distress: fixed-effects model analysis in Japan. J Occup Health.

[34] Labrague L, De Los Santos JAA (2020). COVID-19 anxiety among front-line nurses: Predictive role of organisational support, personal resilience and social support. J Nurs Manag.

[35] Evanoff B, Strickland J, Dale A, Hayibor L, Page E, Duncan J, Kannampallil T, Gray DL (2020). Work-Related and Personal Factors Associated With Mental Well-Being During the COVID-19 Response: Survey of Health Care and Other Workers. J Med Internet Res.

[36] McGinty E, Presskreischer R, Han H, Barry CL (2020). Psychological Distress and Loneliness Reported by US Adults in 2018 and April 2020. JAMA.

[37] Chen Q, Liang M, Li Y, Guo J, Fei D, Wang L, He Li, Sheng C, Cai Y, Li X, Wang J, Zhang Z (2020). Mental health care for medical staff in China during the COVID-19 outbreak. Lancet Psychiatry.

[38] Ryan R, Deci E (2018). Self-Determination Theory Basic Psychological Needs in Motivation, Development, and Wellness.

[39] Ramaci T, Pellerone M, Ledda C, Rapisarda V (2017). Health promotion, psychological distress, and disease prevention in the workplace: a cross-sectional study of Italian adults. Risk Manag Healthc Policy.

[40] Ruiz-Frutos C, Ortega-Moreno M, Allande-Cussó R, Ayuso-Murillo D, Domínguez-Salas S, Gómez-Salgado J (2021). Sense of coherence, engagement, and work environment as precursors of psychological distress among non-health workers during the COVID-19 pandemic in Spain. Saf Sci.

[41] Schaufeli W, Bakker A (2006). UWES Utrecht work engagement scale. Preliminary manual Version 1. APA PsychNet.

[42] Seppälä P, Mauno S, Feldt T, Hakanen J, Kinnunen U, Tolvanen A, Schaufeli W (2008). The Construct Validity of the Utrecht Work Engagement Scale: Multisample and Longitudinal Evidence. J Happiness Stud.

[43] Pejtersen J, Kristensen TS, Borg V, Bjorner JB (2010). The second version of the Copenhagen Psychosocial Questionnaire. Scand J Public Health.

[44] Goldberg D, Gater R, Sartorius N, Ustun T, Piccinelli M, Gureje O, Rutter C (1997). The validity of two versions of the GHQ in the WHO study of mental illness in general health care. Psychol Med.

[45] Schunck R, Perales F (2017). Within- and Between-cluster Effects in Generalized Linear Mixed Models: A Discussion of Approaches and the Xthybrid command. The Stata Journal.

[46] Schunck R (2013). Within and between Estimates in Random-Effects Models: Advantages and Drawbacks of Correlated Random Effects and Hybrid Models. The Stata Journal.

[47] Selya AS, Rose JS, Dierker LC, Hedeker D, Mermelstein RJ (2012). A Practical Guide to Calculating Cohen's f(2), a Measure of Local Effect Size, from PROC MIXED. Front Psychol.

[48] Lorah J (2018). Effect size measures for multilevel models: definition, interpretation, and TIMSS example. Large-scale Assess Educ.

[49] Hakanen J, Ropponen A, Schaufeli W, De Witte H (2019). Who is Engaged at Work?: A Large-Scale Study in 30 European Countries. J Occup Environ Med.

[50] Hakanen J (2009). Työn imun arviointimenetelmä. Työn imu-menetelmän (Utrecht Work Engage-ment Scale) käyttäminen, validointi ja viitetiedot Suomessa Utrecht Work Engagement Scale: Finnish Manual, Validation and Reference Data.

[51] Freedy J, Saladin M, Kilpatrick D, Resnick H, Saunders B (1994). Understanding acute psychological distress following natural disaster. J Trauma Stress.

[52] Rettie H, Daniels J (2020). Coping and tolerance of uncertainty: Predictors and mediators of mental health during the COVID-19 pandemic. Am Psychol.

[53] Maier C, Laumer S, Weinert C, Weitzel T (2015). The effects of technostress and switching stress on discontinued use of social networking services: a study of Facebook use. Info Systems J.

[54] Salo M, Pirkkalainen H, Koskelainen T (2018). Technostress and social networking services: Explaining users' concentration, sleep, identity, and social relation problems. Info Systems J.

[55] Leidner D, Pan G, Pan S (2009). The role of IT in crisis response: Lessons from the SARS and Asian Tsunami disasters. J Strateg Inf Syst.

[56] Leong C, Pan S, Ractham P, Kaewkitipong L (2015). ICT-Enabled Community Empowerment in Crisis Response: Social Media in Thailand Flooding 2011. JAIS.

[57] Charoensukmongkol P, Phungsoonthorn T (2020). The effectiveness of supervisor support in lessening perceived uncertainties and emotional exhaustion of university employees during the COVID-19 crisis: the constraining role of organizational intransigence. J Gen Psychol.

[58] Hakanen JJ, Peeters MCW, Schaufeli WB (2018). Different types of employee well-being across time and their relationships with job crafting. J Occup Health Psychol.

[59] Christian M, Garza A, Slaughter J (2011). Work engagement: A quantitative review and test of its relations with task and contextual performance. Pers Psychol.

