# Supplementary material for: Professional Social Media Usage and Work Engagement Among Professionals in Finland Before and During the COVID-19 Pandemic: Four-Wave Follow-Up Study
Source: J Med Internet Res. 2021 Jun 15;23(6):e29036. doi: 10.2196/29036 (PMC8208471; doi:10.2196/29036)
Supplement: Multimedia Appendix 1 [file jmir_v23i6e29036_app1.docx]

**Multimedia Appendix 1: Copenhagen Psychosocial Questionnaire II Interpersonal relations and leadership dimension**

Rate the following statements on a scale of 1 (*never/hardly ever*), 2 (*seldom*), 3 (*sometimes*), 4 (*often*), or 5 (*always*):

How often do you get help and support from your colleagues?

How often do you get help and support from your nearest superior?

Is there a good atmosphere between you and your colleagues?

Do you feel part of a community at your place of work?
