# Supplementary material for: Professional Social Media Usage and Work Engagement Among Professionals in Finland Before and During the COVID-19 Pandemic: Four-Wave Follow-Up Study
Source: J Med Internet Res. 2021 Jun 15;23(6):e29036. doi: 10.2196/29036 (PMC8208471; doi:10.2196/29036)
Supplement: Multimedia Appendix 2 [file jmir_v23i6e29036_app2.docx]

**Multimedia Appendix 2: Copenhagen Psychosocial Questionnaire II Work organization and job contents dimension**

Rate the following statements on a scale of 1 (*never/hardly ever*), 2 (*seldom*), 3 (*sometimes*), 4 (*often*), or 5 (*always*):

Can you influence on the amount of work assigned to you?

Do you have any influence on what you do at work?

Is your work meaningful?

Can you use your skills or expertise at work?
