# Supplementary material for: Professional Social Media Usage and Work Engagement Among Professionals in Finland Before and During the COVID-19 Pandemic: Four-Wave Follow-Up Study
Source: J Med Internet Res. 2021 Jun 15;23(6):e29036. doi: 10.2196/29036 (PMC8208471; doi:10.2196/29036)
Supplement: Multimedia Appendix 3 [file jmir_v23i6e29036_app3.docx]

**Multimedia Appendix 3: Correlation matrix**
